# A New Parasiticidal Compound in *T. solium* Cysticercosis

**DOI:** 10.1155/2013/505240

**Published:** 2012-12-20

**Authors:** Romel Hernández-Bello, Galileo Escobedo, Julio Cesar Carrero, Claudia Cervantes-Rebolledo, Charles Dowding, James Frincke, Chris Reading, Jorge Morales-Montor

**Affiliations:** ^1^Departamento de Inmunología, Instituto de Investigaciones Biomédicas, Universidad Nacional Autónoma de México, AP 70228, 04510 México, DF, Mexico; ^2^Departamento de Microbiología, Facultad de Medicina, Universidad Autónoma de Nuevo León, 64460 Monterrey, NL, Mexico; ^3^Unidad de Medicina Experimental, Hospital General de México, 06726 México, DF, Mexico; ^4^Hollis-Eden Pharmaceuticals, Inc., San diego, CA 92121, USA

## Abstract

The effect of 16**α**-bromoepiandrosterone (EpiBr), a dehydroepiandrosterone (DHEA) analogue, was tested on the cysticerci of *Taenia solium*, both *in vitro* and *in vivo*. *In vitro* treatment of *T. solium* cultures with EpiBr reduced scolex evagination, growth, motility, and viability in dose- and time-dependent fashions. Administration of EpiBr prior to infection with *T. solium* cysticerci in hamsters reduced the number and size of developed taenias in the intestine, compared with controls. These effects were associated to an increase in splenocyte proliferation in infected hamsters. These results leave open the possibility of assessing the potential of this hormonal analogue as a possible antiparasite drug, particularly in cysticercosis and taeniosis.

## 1. Introduction

Cysticercosis, caused by the metacestode stage of *Taenia solium*, is a serious health and veterinary problem in many developing countries [1–3]. In humans, *T. solium* cysticerci cause neurocysticercosis, which affects ~50 million people worldwide, and it has been considered as an emergent disease in the United States [4]. *T. solium* also infects pigs, its intermediate host, leading to major economic losses [5, 6].

When humans ingest undercooked contaminated pork meat, the adult worm develops in the small intestine. After two months of asymptomatic infection, this tapeworm starts producing thousands of eggs, that, once released with the stools, can contaminate the environment, infecting pigs (rapidly differentiating into cysticerci mainly in the muscle) and humans (where most severe symptoms are observed due to the presence of cysticerci in the brain) [1, 7].

Thus, maintenance of the parasite's life cycle depends on the adult tapeworm development [8]. In fact, even in communities which do not rear or consume pigs, human neurocysticercosis can be found, because of the presence of a tapeworm carrier [9, 10]. Furthermore, tapeworm development in turn depends on scolex evagination, the initial step through which a single cysticercus becomes an adult parasite with capability of producing infective eggs [11].

Dehydroepiandrosterone (DHEA) is a steroid hormone produced from cholesterol by the adrenal glands, the gonads, adipose tissue, and the brain. It is the most abundant hormone in the human body. In humans and in mammals generally (except for rodents), DHEA is the dominant steroid hormone and precursor of sex steroids and has proved to be an important molecule in the immune responses that could drive resistance against a variety of infections [12, 13]. These infections include intracellular parasites such as *Cryptosporidium parvum* [14] and *Plasmodium falciparum* [15] as well as extracellular parasites such as *E. histolytica* [16], *Schistosoma mansoni* [17], and* Taenia crassiceps* [18], among others. 16-bromoepiandrosterone (EpiBr), a DHEA analogue without significant androgenic activity and also known as HE2000, has shown activity in a number of infectious disease settings, including tuberculosis [19], feline immunodeficiency virus viremia [20], malaria [21], cysticercosis by *T. crassiceps,* and amoebiasis by *E. histolytica* [16]. As it can be seen, direct effects of sex steroids upon helminth parasites (cestodes, nematodes, and trematodes) and protozoan parasites are not unusual. In fact, previous results suggest that these pathogens not only are directly affected by adrenal hormones, but they have also developed several strategies to exploit the host's endocrine microenvironment [22, 23], which include degradation of host proteins as an alternative source of amino acids [24], development of parasitic-sex steroid receptors [25, 26], and cross-activation of signal transduction pathways [27, 28].

 Taking into consideration this information, the aim of the present study was to explore whether EpiBr has direct *in vitro* and *in vivo* modulating effects on *T. solium*. Our results suggest that EpiBr treatment may be used as a new therapeutic approach against natural cysticercosis and taeniosis.

## 2. Materials and Methods

### 2.1. Ethics Statement

Animal care and experimentation practices at the Instituto de Investigaciones Biomédicas are frequently evaluated by the Institute's Animal Care and Use Committee, according to the official Mexican regulations (NOM-062-ZOO-1999). Mexican regulations are in strict accordance with the recommendations in the Guide for the Care and Use of Laboratory Animals of the National Institutes of Health (NIH and The Weatherall Report) of the USA, to ensure compliance with established international regulations and guidelines. The protocol was approved by the Committee on the Ethics of Animal Experiments of the Instituto de Investigaciones Biomédicas (Permit number 2009-16). Pigs sacrifice to obtain parasites was performed under sodium pentobarbital anesthesia, and all efforts were made to minimize suffering. All animals were maintained in a common room under controlled temperature and a 14 h dark/10 h light cycle in the animal facility of the Biological Sciences Building at the Institute of Biomedical Research (IIB).

### 2.2. Obtention of Parasites

*T. solium* cysticerci were dissected from the muscle of infected pigs, which were euthanized at the Veterinary School of the Universidad Nacional Autónoma de México. The method was previously evaluated by the University Animal Care and Use Committee to ensure compliance with international regulations and guidelines. The fibrous capsule that surrounds each parasite was carefully separated with the use of a dissection microscope. Once dissected, cysticerci were placed in tubes containing sterile PBS (1X) supplemented with 100 U/mL of antibiotics fungizone (Gibco, Grand Island, NY) [29]. Samples were centrifuged for 10 min, at 800 g at 4°C, and the supernatant was discarded. Pellets containing cysticerci were incubated in Dulbecco's Modified Eagle Medium (DMEM) without fetal serum supplementation (Gibco, BRL, Rockville, MD). They were then washed by centrifugation 3 times for 10 min at 800 g with DMEM. After the final wash, viable parasites (complete and translucent cystic structures) were counted using a binocular microscope.

### 2.3. *In Vitro* EpiBr Assays

Culture grade 16*α*-bromoepiandrosterone (EpiBr) was obtained from Hollis-Eden Pharmaceuticals. EpiBr was dissolved in corn oil to the desired stock concentration and sterilized by passage through a 0.2 *μ*m millipore filter. For *in vitro* tests against *T. solium* cysticerci, hormone analogue was dissolved in corn oil and AIM-V (free of calf serum and other hormones) culture medium to the desired stock concentration and sterilized by passage through a 0.2 *μ*m millipore filter. The experimental design was as follows: using a 24-well culture plate, six wells were used for untreated controls, six wells were supplemented with the vehicle in which EpiBr was diluted, and six wells were treated with different concentrations of EpiBr. The analogue concentrations used were based on the DHEA concentrations of serum levels found in humans and other species and were chosen to approximate analogue levels *in vivo*. The analogue was administered every other day, based on the time that DHEA (the natural hormone) remains circulating before being metabolised and filtered in the kidney. This assures that there is no toxic effect of the analogue, and the circulating levels are proximate to what we want. Our concentrations were calculated to resemble those observed *in vitro* and going from lower to higher levels. However, we converted the doses to concentrations, in order to be in line with most of the studies that use hormones *in vitro* and report those doses in terms of concentration as micromolar. So, based on these facts, we choose analogue doses to be used in both experimental systems. Concentrations of EpiBr were randomised across the plates. Control cysticerci were treated with the solvent in which EpiBr was diluted, so that a constant volume of solvent (2 mL) was added to each well. Scolex evagination and worm length were daily determined in all cultured cysticerci using an inverted microscope (Olympus, MO21, Tokyo) at 10x and 20x magnification. Worm length was considered as the millimetric addition of scolex, neck, and strobila. Immediately, the new cultures (2 mL in glass tubes) were added with 50 *μ*L of corn oil diluted in medium (control) or 50 *μ*L of several concentrations of EpiBr ranging from 0.1 to 50 *μ*g/mL. Samples of 100 *μ*L were collected from the same tube with a particular dose at 24, 48, and 72 h by ice chilling for 5 min and centrifugation at 150 g for 5 min at 4°C. 

### 2.4. *In Vivo* Infections with *T. solium*

*T. solium* cysticerci were selected according to the macroscopic criteria reported by León-Cabrera and coworkers [30]. Briefly, parasites were dissected from muscle of naturally infected pigs, which were euthanized at the Veterinary School of the Universidad Nacional Autónoma de México, under consent of the University Animal Care and Use Committee to ensure compliance with international regulations and guidelines. The fibrous capsule surrounding each parasite was carefully separated under a dissection microscope. Once separated, cysticerci were placed in tubes containing sterile PBS (1X) supplemented with 100 U/mL of antibiotics fungizone (Gibco, Grand Island, NY). Samples were centrifuged at 800** **g at 4°C for 10** **min, and the supernatant was discarded. Pellets containing cysticerci were placed in Dulbecco**'**s Modified Eagle Medium (DMEM, Gibco, BRL, Rockville, MD) without fetal serum supplementation. Then, they were washed and centrifuged 3 times at 800** **g at 4°C for 10** **min. After the final wash, complete and translucent reddish cysticerci were incubated on 6-well culture plates containing DMEM medium with 25% pig fresh bile supplementation for infectivity test. When the evagination rate was higher than 90%, then parasites were used for subsequent oral infections.

### 2.5. EpiBr Administration

Twenty male golden hamsters (*Mesocricetus auratus*) of 140–160 gr, aging between 8 and 10 weeks, were subcutaneously administered with 2 mg/Kg body weight of EpiBr. Ten were infected with *T. solium* as previously described. Each single dose of EpiBr was diluted in 0.4 mL of saline solution (0.9% NaCl, Baker). Control infected animals (*n* = 10 for *T. solium* infections) received 0.4 mL of saline solution as vehicle. A stress-related additional control group (*n* = 10 for *T. solium* infections) was included in our experiments, which consisted in ten sham injection animals. Hormone analogue and vehicle administration was carried out each other day during four weeks, in order to maintain the same hormonal serum concentration for the entirely time of the experiment. Our results were obtained from two independent experiments performed in similar conditions. Animals were fed with Purine Diet 5015 (Purine, St. Louis, MO) and water *ad libitum* during all the experiment. During animal necropsy, the entire small intestine and liver were dissected and placed on a Petri dish containing sterile PBS (1X) (Sigma-Aldrich, USA). Using a stereoscopic microscope, the lumen of all small intestines was carefully exposed by making a longitudinal cut using sterile dissection scissors. Then, duodenum-anchored parasites were counted and measured with a calibrator. Blood samples were individually collected from all animal groups for posterior serum analysis. Ileum attachment zones where *T. solium* scolices were located were placed in 4% paraformaldehyde (J. T. Baker, México), or Trizol reagent (Invitrogen, Carlsbad, CA) for posterior analysis. Immediately after necropsy, spleen weight was individually recorded. Spleen and liver samples from all animal groups were individually obtained and placed in RPMI (Gibco, BRL, Rockville, MD) supplemented with 10% fetal calf serum (Gibco, BRL, Rockville, MD), or 4% paraformaldehyde (J. T. Baker, México), or Trizol reagent (Invitrogen, Carlsbad, CA) for posterior analysis.

### 2.6. Cell Culture and Lymphoid Proliferation

Total leukocytes and red blood cells were extracted from spleen and mesenteric lymph nodes of all animal groups. After single washing with ACK Lysing Buffer (Invitrogen, USA), total leukocytes were recovered and cultured in 96-well sterile plates (1 × 10^4^ cells/well) containing serum-free RPMI medium (Gibco-BRL), at 37°C in humidified 5% CO_2_ atmosphere for 72 h. After this time, cultured leukocytes from spleen and mesenteric lymph nodes of all animals were exposed to 15 *μ*g/well of freshly extracted *T. solium* total antigen during 48 h. Twenty-four hours before the end of the experiment, 20 *μ*L of AlamarBlue reagent (Biosource International) were added to each culture well. Then, culture plates were frozen at −30°C under darkness and the absorbance was quantified at 570 and 600 nm, using a microplate reader. The 570–600 nm lecture coefficient was employed to assess proliferation index.

### 2.7. Experimental Design and Statistical Analysis

We used a two factorial experiment. Independent variables were (1) treatment (two levels: EpiBr or vehicle); and (2) infection (two levels: Yes, No). The dependent variable was the number and size of parasites. Two *in vivo* experiments were performed, and data were analysed using one-way analysis of variance (ANOVA). When performed, post hoc individual contrasts of group means to test for significant differences were carried out using *t*-tests. Hormone dose-response time curves were estimated in three independent experiments performed with freshly isolated *T. solium* cysticerci. EpiBr was tested at five different doses; each dose was run in triplicate. Differences between groups were estimated by the ANOVA test. Post hoc analysis used was the *t*-test to examine for significant differences. Differences were considered significant when *P* < 0.01. The software Prism 2.01 (GraphPad Software Inc.) was used to calculate probability values.

## 3. Results

### 3.1. *In Vitro* Effect on *T. solium* Cysticerci

**Figure 1:**
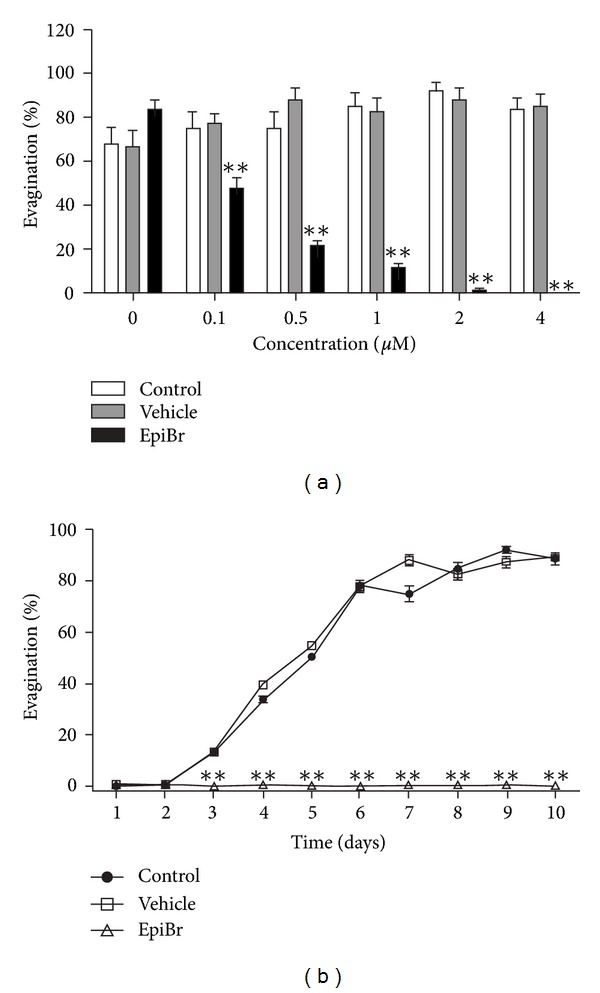
EpiBr decreased scolex evagination of *Taenia solium* in a concentration-dependent pattern (a) maintained along the time (b). In concentration-response curves (panel (a)), cysticerci treated with vehicle are referred to as concentration zero. Data are represented as mean +/− SD. **P < 0.05.

When *T. solium* cysticerci were *in vitro* exposed to EpiBr, a decrease in the scolex evagination was observed in all treated parasites compared to control groups (Figure 1(a)). However, this evagination-inhibiting effect mediated by EpiBr was independent of the tested concentrations (Figure 1(a)). Concomitantly, the evagination-inhibiting effect of EpiBr (0.25 *μ*M) was maintained through all 10 days of *in vitro* culture, reaching its highest response on the third day of culture, in relation to control and vehicle parasites (Figure 1(b)). It is important to mention that viability of evaginated cysticerci was verified daily by means of worm motility in the culture plate, which was constant through all days of *in vitro* culture. Injured parasites were recognized by a progressive internal disorganization: development of opaque areas in the tegument and loss of translucence of the vesicle.

**Figure 2:**
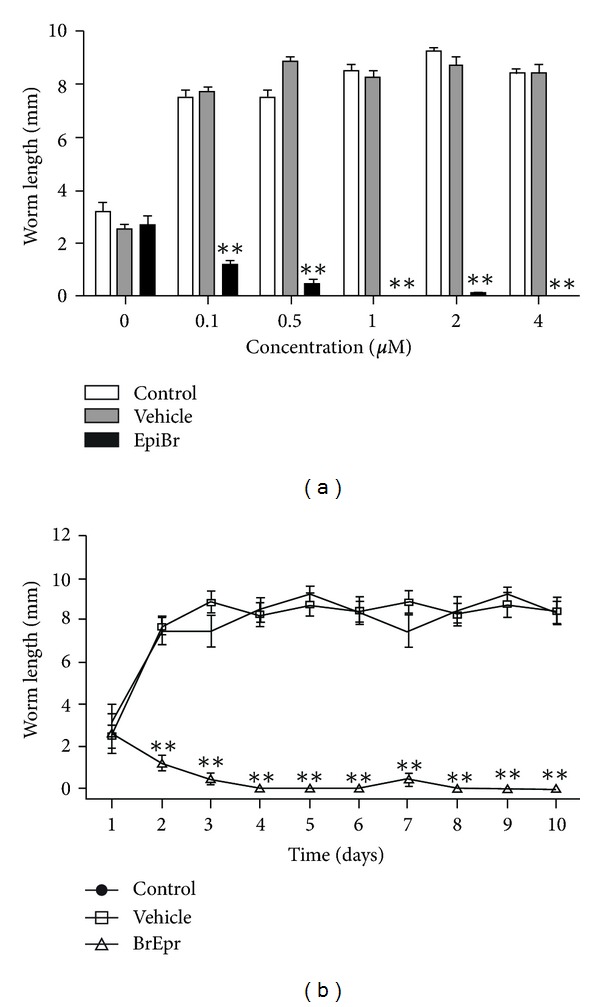
EpiBr decreases worm growth in a concentration-dependent pattern (a), reaching its maximum effect at 4 days of *in vitro* during the whole culture time (b). EpiBr-treated parasites were motile and undamaged on the culture plate. Worm length was considered as the addition (mm) of scolex, neck, and strobila. In the concentration-response curves (panel (a)), cysticerci treated with vehicle are referred to as concentration zero. Data are represented as mean +/− SD. **P < 0.05.

EpiBr also affected *in vitro* worm growth. From the lowest concentration (0.1 *μ*M), EpiBr inhibited worm length on day 10 (measured as the addition of scolex, neck, and strobila of the developing parasite) with respect to the control group and reached a plateau (Figure 2(a)). In addition, the *T. solium* worm gradually decreased up in response to 0.5 *μ*M of EpiBr (Figure 2(b)). Differentiated worms in absence of hormones or vehicle stimulus had a spontaneous development, reaching their maximum length (8.5 mm) at 3 day in culture. Once again, in the presence of EpiBr, no worm differentiation was observed with 2.0 *μ*M along all the time of *in vitro* culture.

### 3.2. *In Vivo* Effect on *T. solium* Infection

**Figure 3:**
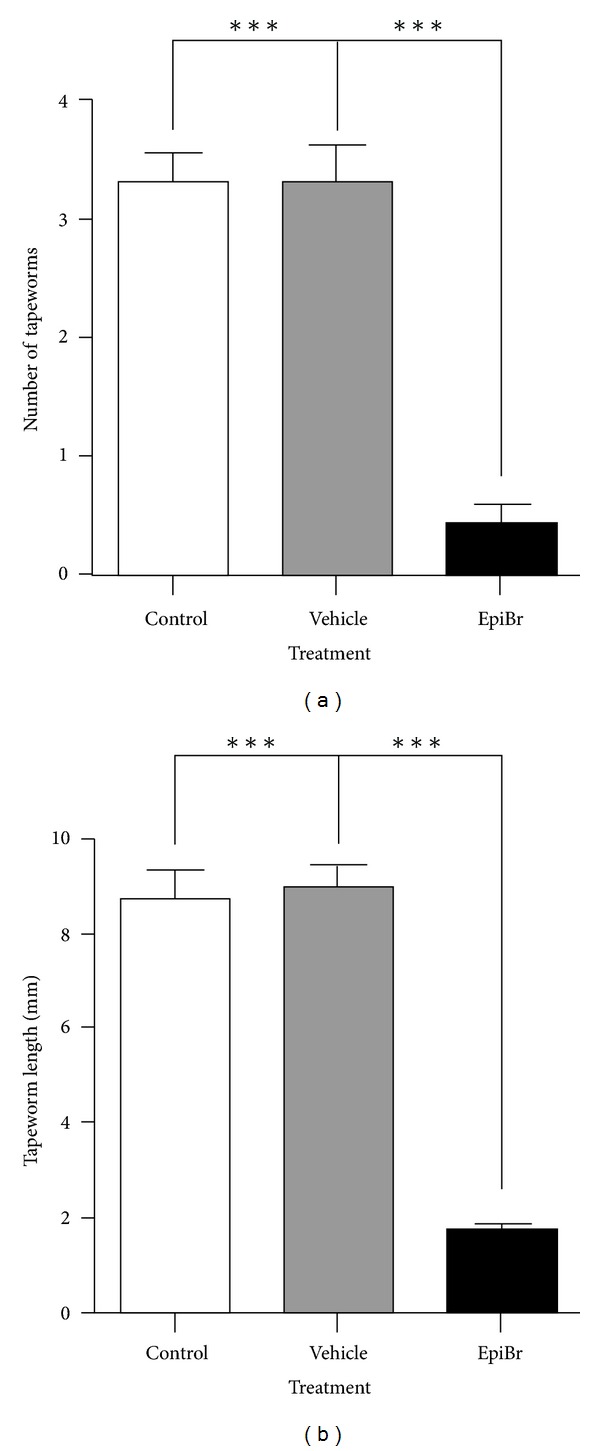
EpiBr decreases both parasite load and tapeworm length in *T. solium* cysticerci orally infected hamsters. (a) Administration of BrEpi significantly diminished the number of intestinally attached adult tapeworms by 80–87%, with respect to both control and vehicle infected groups. (b) Parasites exposed to constant concentrations of BrEpi showed total length reduction of fourfold. These parasites seemed as undifferentiated scolices with no develop of neck and strobila, compared to those tapeworms from control and vehicle groups with well-differentiated structures. Tapeworm length was determined as the longitudinal sum of scolex, neck, and strobila. Nonmanipulated infected hamsters were denominated as control, meanwhile comparative lines represent significant differences when *P* < 0.05. Results are presented as mean +/− standard deviation.

After 15 days after infection, EpiBr treatment significantly reduced the number of intestinally anchored *T. solium* tapeworms by 80–87% (Figure 3(a)). Vehicle-treated and control-infected hamsters showed between three and four viable parasites (Figure 3(a)). It is important to remark that all found tapeworms were strongly attached to the duodenum zone. As expected, tapeworms from vehicle-treated and control-infected hamsters grew up more than 8.8 ± 1.6 mm (Figure 3(b)). In contrast, parasite from EpiBr-treated hamsters did not develop more than 1.7 ± 0.3 mm in length (Figure 3(b)), showing besides poorly differentiated scolices. Thus, steady concentrations of EpiBr exerted a protective role against the *T. solium* intestinal infection, diminishing both the number of attached parasites and their development.

**Figure 4:**
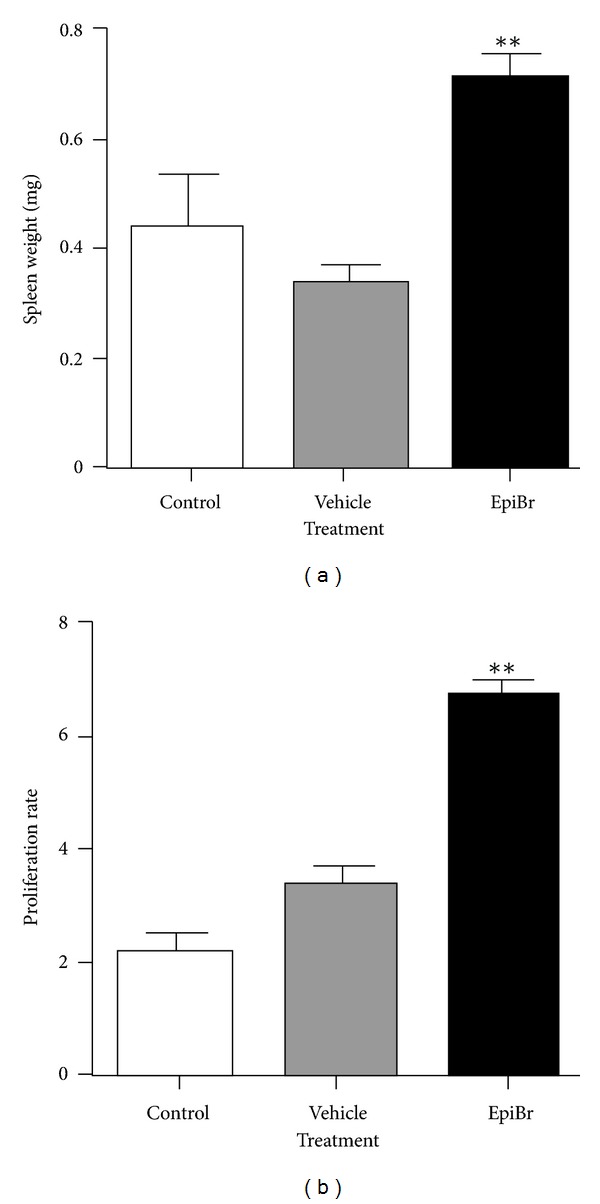
EpiBr administration increases proliferation rate of parasite specific leukocytes from spleen. (a) EpiBr-treated animals showed a tendency to increase spleen weight, with respect to control- and vehicle-treated animals. (b) Spleen leukocytes from EpiBr-treated, vehicle, and control infected hamsters were *in vitro* cultured in presence of *T. solium* total antigen. As a consequence of the *in vivo* exposition to EpiBr, the leukocyte proliferation index in presence of parasitic antigen was augmented by 3.5- and 2.7-fold, compared to immune cells from both control- and vehicle-treated hamsters, respectively. Results are presented as mean +/− standard deviation. **P < 0.05 versus the other groups.

To assess the possible mechanism involved in EpiBr protective actions during infection, spleens from all animal groups were weighed and splenic leukocytes assayed for antigen-specific proliferation (Figure 4). Significant differences were observed among the spleen weight from EpiBr-treated hamsters, compared with vehicle-treated animals and control-infected hamsters (Figure 4(a)), where hamsters exposed to EpiBr increase spleen weight with respect to their infected control littermates. Furthermore, *in vivo* EpiBr treatment clearly increased proliferation *in vitro* of *T. solium* antigen-specific leukocytes by 3.5-fold compared to both infected control groups (Figure 4(b)). This result suggests that EpiBr should protect hamsters from *T. solium* infection through promotion of a local mucosal antiparasite immune response.

## 4. Discussion

It has been reported that exogenous DHEA administration upregulates the immune system, specifically the cellular immune response, by increasing the natural killer cell number and function [13]. Our previous findings do not support this notion, since IL-2 mRNA levels do not change in response to DHEA treatment. The lack of effect of DHEA on cytokine mRNA but its dramatic effect *in vivo* on parasite load and parasite reproduction and *in vitro* on survival supports the hypothesis that DHEA analogue exerts its protective properties via direct effects on the parasite. To the best of our knowledge, this effect is consistent with the known effects of DHEA on the survival of other parasites, both metazoan [17, 31] and protozoan [16].

For instance, it has been suggested that in human schistosomiasis, DHEA is the cause of the puberty-associated drop in susceptibility [32]. This idea has been reinforced by experiments in which treatment of mice with the bloodstream form of DHEA, DHEA-S (DHEA-sulfate), protected them from infection with *S. mansoni *[17] and mice against *T. crassiceps* infection [18]. 

Other findings in mice of a decrease in DHEA levels as infection progresses agree with previous results in a *S. mansoni*-baboon model, in which baboons with primary infections showed decreasing levels of DHEA as the infection progressed, compared with uninfected and reexposed baboons [31].

The protective effect of DHEA has also been demonstrated in parasitic infections like *T. cruzi*, where increase the levels of lytic antibodies and to reduce *T. cruzi* parasitemia in rats [33] or in the protozoan parasite *C. parvum*, where significantly reduced both the shedding of fecal oocysts and parasite colonisation of the ileum [34, 35]. On the other hand, 16-bromoepiandrosterone (EpiBr), a DHEA analogue without significant androgenic activity and also known as HE2000, has activity in a number of infectious disease settings [16, 18–20]. Here, we explored whether EpiBr has direct *in vitro* and *in vivo* modulating effects on *T. solium* and the immunomodulating effects on *E. histolytica* reproduction, growth, viability, and infectivity.

The *in vitro* DHEA treatment of *E. histolytica* trophozoites also reduced the growth and viability of this parasite. The effects of DHEA were associated with the inhibition of G6PD activity [16]. Also, DHEA is known to exert antimalarial protection, via the enhanced opsonisation and phagocytosis of rings, the early forms of this parasite [36, 37]. Our results confirm and extend the notion that DHEA is a strong parasiticidal agent, since *in vitro* EpiBr treatment of *T. solium* remarkably reduced the reproduction rate and viability of cysticerci. Also, in our present experiments, the effects of EpiBr significantly reduced the parasite burden in males. Finally, our results support and extend the notion that EpiBr is a potentially useful treatment against a large variety of parasitic diseases. The fact that EpiBr interferes with the development of *T. solium* cysticerci may be applied to the development of future therapeutic protocols against this parasite that affects pigs and humans. 

DHEA is a hormone able to inhibit the *in vitro *growth of numerous parasites, including *T. crassiceps*, a murine cestode, and *E. histolytica*, the causal agent of amoebiasis in human [16, 18]. Such results, in addition to the benefits discovered for this hormone on neuroprotection, anxiety, depression, schizophrenia, dementia, some neoplasias such as breast cancer, diabetes, and numerous inflammatory disorders [38–40], have raised discussion about the possibility of using the DHEA analogue, EpiBr, for therapeutic purposes, including the control of parasitic diseases. Thus, analogs lacking androgenic activity but maintaining the other biological activities of the natural steroid have been designed. In this study, we provide evidence demonstrating the ability of the analog EpiBr for inhibiting the establishment and growth of *T. solium*. *In vitro*, the effect of the analogue on the parasite was observed in several parameters of cell and organic functionality, affecting the establishment and growth and survival in general. These effects were similar to those previously reported by our group regarding the *in vitro* effect of DHEA on both *Taenia crassiceps* and *Entamoeba histolytica* [16–18]. Our results are in agreement with similar inhibitory effect shown for the analog on the proliferation of *P. falciparum* and *P. berghei* parasites *in vitro* [21]. 

The *in vitro* inhibitory effects of EpiBr on *T. solium* were also extended to the *in vivo* infections. Thus, treatment with the analog decreased the *T. solium *cysticerci evagination and growth up to 80–87% in hamsters. 

Since EpiBr enhanced splenocyte proliferation, we suggest that its *in vivo* protective activity was likely due, at least in part, to a possible immunomodulatory effect on the host by EpiBr. The latter theory could be in fact involved as EpiBr has anti-inflammatory properties and it is also able to induce cellular immunity that may aid the control of infections [19, 41, 42]. Our results *in vivo* are also in agreement with previous studies supporting a potential pharmacological use of EpiBr to treat infections such as tuberculosis, AIDS, and malaria [19, 21, 41, 42]. Thus, administration of intramuscular HE2000 in patients with *P. falciparum* malaria resulted in a 50% reduction of parasitemia with notably improve in symptomatology and mild adverse events [43]. 

## 5. Conclusion

We have demonstrated that EpiBr is a promising new compound that can be used in cysticercosis, taeniosis. The results of this study leave open the possibility of assessing the potential of this analog of DHEA as an antiparasitic drug, and in particular against human/pig cysticercosis, taeniosis, major health public problems by parasites in developing countries.

## References

[1] Nash TE, Singh G, White AC (2006). Treatment of neurocysticercosis: current status and future research needs. *Neurology*.

[2] Garcia HH, Del Brutto OH (2005). Neurocysticercosis: updated concepts about an old disease. *Lancet Neurology*.

[3] Flisser A, Sarti E, Lightowlers M, Schantz P (2003). Neurocysticercosis: regional status, epidemiology, impact and control measures in the Americas. *Acta Tropica*.

[4] White AC (1997). Neurocysticercosis: a major cause of neurological disease worldwide. *Clinical Infectious Diseases*.

[5] Fan PC, Chung WC (1997). Sociocultural factors and local customs related to taeniasis in east Asia. *The Kaohsiung Journal of Medical Sciences*.

[6] Gonzalez AE, Gavidia C, Falcon N (2001). Protection of pigs with cysticercosis from further infections after treatment with oxfendazole. *American Journal of Tropical Medicine and Hygiene*.

[7] Pawlowski Z, Allan J, Sarti E (2005). Control of *Taenia solium* taeniasis/cysticercosis: from research towards implementation. *International Journal for Parasitology*.

[8] Behravesh CB, Mayberry LF, Bristol JR (2008). Population-based survey of taeniasis along the United States-Mexico border. *Annals of Tropical Medicine and Parasitology*.

[9] Moore AC, Lutwick LI, Schantz PM (1995). Seroprevalence of cysticercosis in an orthodox Jewish community. *American Journal of Tropical Medicine and Hygiene*.

[10] Schantz PM, Moore AC, Munoz JL (1992). Neurocysticercosis in an Orthodox Jewish community in New York City. *New England Journal of Medicine*.

[11] Rabiela MT, Hornelas Y, García-Allan C, Rodríguez-del-Rosal E, Flisser A (2000). Evagination of *Taenia solium* cysticerci: a histologic and electron microscopy study. *Archives of Medical Research*.

[12] Allolio B, Arit W (2002). DHEA treatment: myth or reality?. *Trends in Endocrinology and Metabolism*.

[13] Loria RM, Padgett DA (1998). Control of the immune response by DHEA and its metabolites. *Rinsho Byori*.

[14] Rasmussen KR, Healey MC (1992). Dehydroepiandrosterone-induced reduction of *Cryptosporidium parvum* infections in aged Syrian golden hamsters. *Journal of Parasitology*.

[15] Kurtis JD, Mtalib R, Onyango FK, Duffy PE (2001). Human resistance to *Plasmodium falciparum* increases during puberty and is predicted by dehydroepiandrosterone sulfate levels. *Infection and Immunity*.

[16] Carrero JC, Cervantes C, Moreno-Mendoza N, Saavedra E, Morales-Montor J, Laclette JP (2006). Dehydroepiandrosterone decreases while cortisol increases *in vitro* growth and viability of *Entamoeba histolytica*. *Microbes and Infection*.

[17] Fallon PG, Richardson EJ, Jones FM, Dunne DW (1998). Dehydroepiandrosterone sulfate treatment of mice modulates infection with *Schistosoma mansoni*. *Clinical and Diagnostic Laboratory Immunology*.

[18] Vargas-Villavicencio JA, Larralde C, Morales-Montor J (2008). Treatment with dehydroepiandrosterone *in vivo* and *in vitro* inhibits reproduction, growth and viability of *Taenia crassiceps* metacestodes. *International Journal for Parasitology*.

[19] Hernández-Pando R, Aguilar-Leon D, Orozco H (2005). 16*α*-Bromoepiandrosterone restores T helper cell type 1 activity and accelerates chemotherapy-induced bacterial clearance in a model of progressive pulmonary tuberculosis. *Journal of Infectious Diseases*.

[20] Pedersen NC, North TW, Rigg R (2003). 16*α*-Bromo-epiandrosterone therapy modulates experimental feline immunodeficiency virus viremia: initial enhancement leading to long-term suppression. *Veterinary Immunology and Immunopathology*.

[21] Freilich D, Ferris S, Wallace M (2000). 16*α*-bromoepiandrosterone, a dehydroepiandrosterone (DHEA) analogue, inhibits *Plasmodium falciparum* and *Plasmodium berghei* growth. *American Journal of Tropical Medicine and Hygiene*.

[22] Escobedo G, Roberts CW, Carrero JC, Morales-Montor J (2005). Parasite regulation by host hormones: an old mechanism of host exploitation?. *Trends in Parasitology*.

[23] Damian RT (1997). Parasite immune evasion and exploitation: reflections and projections. *Parasitology*.

[24] Shibayama M, Serrano-Luna JDJ, Rojas-Hernández S, Campos-Rodríguez R, Tsutsumi V (2003). Interaction of secretory immunoglobulin A antibodies with Naegleria fowleri trophozoites and collagen type I. *Canadian Journal of Microbiology*.

[25] Remoué F, Mani JC, Pugnière M, Schacht AM, Capron A, Riveau G (2002). Functional specific binding of testosterone to *Schistosoma haematobium* 28-kilodalton glutathione S-transferase. *Infection and Immunity*.

[26] Konrad C, Kroner A, Spiliotis M, Zavala-Góngora R, Brehm K (2003). Identification and molecular characterisation of a gene encoding a member of the insulin receptor family in *Echinococcus multilocularis*. *International Journal for Parasitology*.

[27] Spiliotis M, Konrad C, Gelmedin V (2006). Characterisation of EmMPK1, an ERK-like MAP kinase from *Echinococcus multilocularis* which is activated in response to human epidermal growth factor. *International Journal for Parasitology*.

[28] Brehm K, Spiliotis M (2008). The influence of host hormones and cytokines on echinococcus multilocuiaris signalling and development. *Parasite*.

[29] Esch GW, Smyth JD (1976). Studies on the *in vitro* culture of *Taenia crassiceps*. *International Journal for Parasitology*.

[30] León-Cabrera S, Cruz-Rivera M, Mendlovic F (2009). Standardization of an experimental model of human taeniosis for oral vaccination. *Methods*.

[31] Morales-Montor J, Newhouse E, Mohamed F, Baghdadi A, Damian RT (2001). Altered levels of hypothalamic-pituitary-adrenocortical axis hormones in baboons and mice during the course of infection with *Schistosoma mansoni*. *Journal of Infectious Diseases*.

[32] Fulford AJC, Webster M, Ouma JH, Kimani G, Dunne DW, Fulford T (1998). Puberty and age-related changes in susceptibility to schistosome infection. *Parasitology Today*.

[33] Dos Santos CD, Alonso Toldo MP, Do Prado JC (2005). *Trypanosoma cruzi*: the effects of dehydroepiandrosterone (DHEA) treatment during experimental infection. *Acta Tropica*.

[34] Rasmussen KR, Martin EG, Healey MC (1993). Effects of dehydroepiandrosterone in immunosuppressed rats infected with *Cryptosporidium parvum*. *Journal of Parasitology*.

[35] Rasmussen KR, Healey MC, Cheng L, Yang S (1995). Effects of dehydroepiandrosterone in immunosuppressed adult mice infected with *Cryptosporidium parvum*. *Journal of Parasitology*.

[36] Ayi K, Giribaldi G, Skorokhod A, Schwarzer E, Prendergast PT, Arese P (2002). 16*α*-bromoepiandrosterone, an antimalarial analogue of the hormone dehydroepiandrosterone, enhances phagocytosis of ring stage parasitized erythrocytes: a novel mechanism for antimalarial activity. *Antimicrobial Agents and Chemotherapy*.

[37] Safeukui I, Mangou F, Malvy D (2004). *Plasmodium berghei*: dehydroepiandrosterone sulfate reverses chloroquino-resistance in experimental malaria infection; correlation with glucose 6-phosphate dehydrogenase and glutathione synthesis pathway. *Biochemical Pharmacology*.

[38] Maninger N, Wolkowitz OM, Reus VI, Epel ES, Mellon SH (2009). Neurobiological and neuropsychiatric effects of dehydroepiandrosterone (DHEA) and DHEA sulfate (DHEAS). *Frontiers in Neuroendocrinology*.

[39] Couillard S, Labrie C, Bélanger A, Candas B, Pouliot F, Labrie F (1998). Effect of dehydroepiandrosterone and antiestrogen EM-800 on growth of human ZR-75-1 breast cancer xenografts. *Journal of the National Cancer Institute*.

[40] Brignardello E, Runzo C, Aragno M (2007). Dehydroepiandrosterone administration counteracts oxidative imbalance and advanced glycation end product formation in type 2 diabetic patients. *Diabetes Care*.

[41] Stickney DR, Noveljic Z, Garsd A, Destiche DA, Frincke JM (2007). Safety and activity of the immune modulator HE2000 on the incidence of tuberculosis and other opportunistic infections in AIDS patients. *Antimicrobial Agents and Chemotherapy*.

[42] Reading C, Dowding C, Schramm B (2006). Improvement in immune parameters and human immunodeficiency virus-1 viral response in individuals treated with 16*α*-bromoepiandrosterone (HE2000). *Clinical Microbiology and Infection*.

[43] Frincke JM, Stickney DR, Onizuka-Handa N (2007). Reduction of parasite levels in patients with uncomplicated malaria by treatment with HE2000. *American Journal of Tropical Medicine and Hygiene*.

